# Electrochemotherapy of Primary Colon Rectum Cancer and Local Recurrence: Case Report and Prospective Analysis

**DOI:** 10.3390/jcm11102745

**Published:** 2022-05-12

**Authors:** Daniela Rega, Vincenza Granata, Antonella Petrillo, Ugo Pace, Massimiliano Di Marzo, Roberta Fusco, Valeria D’Alessio, Guglielmo Nasti, Carmela Romano, Antonio Avallone, Vincenzo Ravo, Fabiana Tatangelo, Piera Maiolino, Raffaele Palaia, Francesco Izzo, Paolo Delrio

**Affiliations:** 1 Division of Colorectal Surgery, Istituto Nazionale Tumori IRCCS Fondazione Pascale—IRCCS di Napoli, 80131 Naples, Italy; d.rega@istitutotumori.na.it (D.R.); u.pace@istitutotumori.na.it (U.P.); m.dimarzo@istitutotumori.na.it (M.D.M.); p.delrio@istitutotumori.na.it (P.D.); 2 Division of Radiology, Istituto Nazionale Tumori IRCCS Fondazione Pascale—IRCCS di Napoli, 80131 Naples, Italy; a.petrillo@istitutotumori.na.it; 3 Italian Society of Medical and Interventional Radiology (SIRM), SIRM Foundation, Via della Signora 2, 20122 Milan, Italy; 4 IGEA SpA Medical Division-Oncology, Via Casarea 65, Casalnuovo di Napoli, 80013 Napoli, Italy; r.fusco@igeamedical.com (R.F.); v.dalessio@igeamedical.com (V.D.); 5 Division of Abdominal Medical Oncology, Istituto Nazionale Tumori IRCCS Fondazione Pascale—IRCCS di Napoli, 80131 Naples, Italy; g.nasti@istitutotumori.na.it (G.N.); c.romano@istitutotumori.na.it (C.R.); a.avallone@istitutotumori.na.it (A.A.); 6 Division of Abdominal Radiotherapy, Istituto Nazionale Tumori IRCCS Fondazione Pascale—IRCCS di Napoli, 80131 Naples, Italy; v.ravo@istitutotumori.na.it; 7 Division of Pathological Anatomy, Istituto Nazionale Tumori IRCCS Fondazione Pascale—IRCCS di Napoli, 80131 Naples, Italy; f.tatangelo@istitutotumori.na.it; 8 Division of Pharmacy, Istituto Nazionale Tumori IRCCS Fondazione Pascale—IRCCS di Napoli, 80131 Naples, Italy; p.maiolino@istitutotumori.na.it; 9 Division of Hepatobiliary Surgery, Istituto Nazionale Tumori IRCCS Fondazione Pascale—IRCCS di Napoli, 80131 Naples, Italy; r.palaia@istitutotumori.na.it (R.P.); f.izzo@istitutotumori.na.it (F.I.)

**Keywords:** electrochemotherapy, endoscopic treatment, minimally invasive, colorectal cancer

## Abstract

Purpose: Surgery, radiotherapy, and oncological treatment (chemotherapy and antineoplastic antibodies) are standard treatments of rectal cancer. ECT has shown its effectiveness and suitability in deep solid tumors conducted in both preclinical and clinical studies. We show here an update and preliminary results with locally advanced rectum cancer (LARC) treated with ECT. Methods: Two patients with major clinical response to restaging after neoadjuvant treatment for LARC were subjected to ECT 12 weeks after completing chemo-radiation therapy. One patient was subjected to ECT on a colorectal local recurrence formed after neoadjuvant treatment for LARC and surgery. Computed Tomography and Magnetic Resonance Imaging were used to assess ECT response. Results: The results showed stable disease in two of the three patients treated, while one patient achieved a complete response. The local control of disease is maintained in the patient follow-up. For each patient, a reduction in pain was observed and for the patient with local recurrence, a reduction in bleeding present before ECT was also achieved. Conclusion: Preliminary results showed that ECT is a safe and effective treatment in patients with a major clinical response or local recurrence after neoadjuvant therapy for LARC and allows a reduction in pain and bleeding with a consequent improvement to quality of life.

## 1. Introduction

Every year, about 1.4 million new cases of colorectal cancer (CRC) are registered worldwide [1], and most of these cases are in older patients (65 years and above) [2]. Standard treatment in patients with locally advanced rectal cancer (LARC) is represented by surgery, radiotherapy, and oncological treatment (chemotherapy and antineoplastic antibodies). New compounds that could potentially be active against colorectal cancer have been synthesized [3].

Unfortunately, between 2.6% and 32% of cases relapse and without adequate surgical treatment, less than 5% of patients survive at 5 years. This percentage can rise to 60% (at 5 years) without radical treatment of the relapse [4]. Survival increases if surgery is associated with adjuvant integrated and intraoperative radiotherapy (+/− chemotherapy) [5,6,7,8].

A high percentage (up to 25%) of colorectal cancer patients have metastases at diagnosis [9]. Although surgery is the gold standard for CLM, only 20% of patients are eligible for surgery [9]. Patients with unresectable liver metastases at diagnosis are typically treated with a combination of surgical and non-surgical procedures such as radiothermal ablation, cryoablation, irreversible electroporation, and portal embolization unless patients had some bilobar involvement or previous liver surgery [10,11,12,13,14,15,16,17]. The aim is to reduce the tumor mass and obtain radicality with subsequent surgery, or to limit the progression of the tumor [10].

Moreover, local treatment such as reversible electroporation could be used to reduce the lesion size before the surgery. Electroporation (EP) is a physical phenomenon whereby if adequate voltage is applied, the membrane becomes permeable, enabling entry into the cell of molecules that are not, or are poorly, permeable. The EP technique used alone as Irreversible Electroporation (IRE), or in combination with anticancer drugs (Electrochemotherapy, ECT), has proven to be effective in the treatment of primary and metastatic tumors. Compared to other techniques such as Radio Frequencies (RF) and Microwave (Microwave) Radiotherapy (RX), the treatment of EP is not associated with a variation in the temperature of the exposed tissue. Therefore, the treatment can also be completed in the vicinity of noble structures (vessels and nerves) without risk of complication. ECT is a locoregional anti-tumor therapy that combines a low dose of chemotherapy with high-intensity electric pulses to induce cell membrane electroporation and, consequently, to locally enhance drug delivery into tumor cells. Bleomycin and cisplatin are the two most used drugs; cisplatin is usually injected intratumorally and bleomycin is injected intratumorally or systemically depending on the dimension and number of lesions. Bleomycin intravenous infusion is the most common administration [18,19]. After the first electrochemotherapy clinical trials [18,19,20,21,22,23], the Standard Operating Procedures for ECT with the CLINIPORATOR™ Device (IGEA S.p.A., Carpi, Italy) were defined [23,24]. ECT clinical effectiveness and its safety profile have been confirmed in several studies [23,24,25,26,27,28], showing no serious adverse effects and limited side effects on normal tissues. Moreover, studies have shown that ECT with bleomycin is safe and improves outcomes for the treatment of squamous cell carcinoma of the head and neck when compared to bleomycin therapy alone [29,30].

ECT has shown its effectiveness and suitability in deep solid tumors conducted in both preclinical and clinical studies [31,32,33,34,35,36,37,38,39,40,41]. ECT was confirmed to be safe and free of intraoperative or postoperative serious adverse events [1,37].

Recently, a phase I clinical trial on endoscopic electrochemotherapy for advanced esophageal cancer was conducted in six patients using intravenous bleomycin (https://clinicaltrials.gov/ct2/show/NCT04649372, accessed on 10 April 2021). The treatment procedure is similar to standard endoscopic colorectal examination (therapeutic colonoscopy) with the added element of an intravenous injection of bleomycin followed by the delivery of electric pulses (each one less than 1 msec in duration). The pulses are endoscopically delivered directly to the tumor mass. The entire procedure is minimally invasive. A successful treatment will cause the tumor to shrink in size in the weeks following the procedure.

The objective of this study is to assess the preliminary finding of ECT used in downsizing locally advanced rectal tumors prior to the intended curative surgery. Multiple needles in a variable geometry and fixed geometry were used to perform the treatment and safety and feasibility for each procedure was considered.

In this study, we present a prospective analysis of two LARC patients with a major clinical response after neoadjuvant treatment at restaging. Patients were subjected to ECT 12 weeks after completing neoadjuvant treatment, and one patient was treated with ECT for local recurrence after surgery. Attention was given to the feasibility and safety of the treatment.

## 2. Patients and Methods

### 2.1. Patients with Primary Cancer

Three LARC patients were treated with long course radiotherapy with concomitant chemotherapy in a neoadjuvant regimen before local excision (LE). Long course radiotherapy consists of 45–50 Gy (in fractions of 1.8–2 Gy), with simultaneous 5-fluorouracil administration. Standard fractions of 1.8 Gy/day to the reference point were given, 5 times a week up to a total dose of 50.4 Gy in 28 fractions [42]. Each subject received the standard treatment with capecitabine at a dose of 825 mg/m2 twice daily, 5 days a week, for 5 weeks.

A 3-field technique (one posterior–anterior and two lateral fields) or IMRT (Intensity Modulated Radiation Therapy) were used for external radiation therapy.

Electrochemotherapy was performed according to ESOPE guidelines. Shortly after the induction of systemic anesthesia, bleomycin (15,000 IU BLM/m^2^) was injected intravenously. Eight minutes after, electric pulses were delivered to the lesion with the CLINIPORATOR™ (IGEA Ltd., Modena, Italy) with the insertion of appropriate CE certificate electrodes (STINGER or VGD applied part by IGEA Ltd., Modena, Italy). The procedure was completed within 40 min from the end of the bleomycin injection [23]. Computed Tomography (CT) and Magnetic Resonance Imaging (MRI) were used to assess ECT response [6,43,44,45,46,47,48,49,50,51,52,53,54]. In Table 1, case descriptions and results are summarized.

### 2.2. First Case of LARC Description

**Figure 1:**
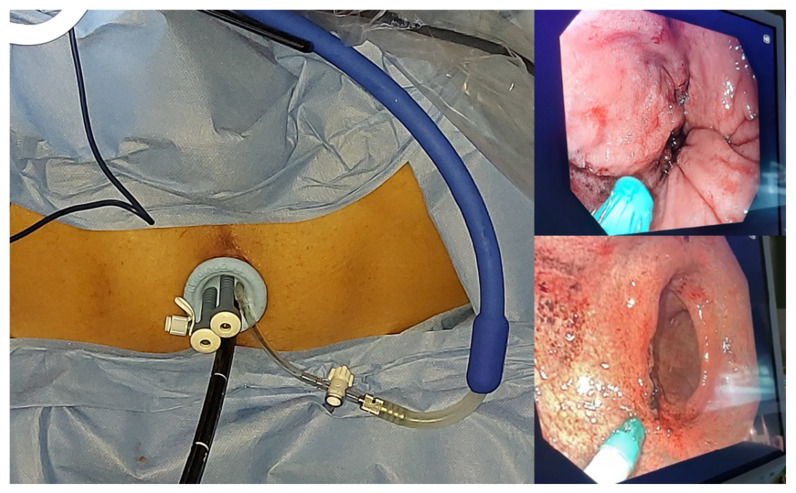
Trans-anal laparoscopic approach with a SILS port for ECT treatment on a lesion of the endorectal mucosa at 6 cm from the anal canal using an expandible divergent electrode with 10 degrees of divergence, a length of 20 cm, and a 5 mm shaft.

A 75-year-old male with a major clinical response to restaging was submitted to ECT treatment 12 weeks after chemo-radiation therapy. A trans-anal laparoscopic approach with a single-incision laparoscopic surgery (SILS) port was used to perform the ECT (Figure 1) on a lesion of the endorectal mucosa at 6 cm from the anal canal. An expandable divergent electrode with 10 degrees of divergence, a length of 20 cm, and a 5 mm shaft (STINGER IG0E821) was used.

### 2.3. Second Case LARC Description

**Figure 2:**
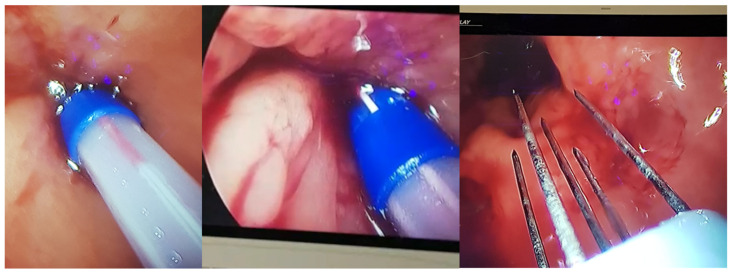
ECT with a trans-anal laparoscopic approach and a SILS port on the primary rectal tumor with semi-annular morphology and extension from hours to 2 to 5 using an expandable divergent electrode with 10 degrees of divergence, a length of 20 cm, and a 5 mm shaft.

A 66-year-old male with a major clinical response to restaging was submitted to ECT treatment 12 weeks after chemo-radiation therapy. ECT with a trans-anal laparoscopic approach and a SILS port (Figure 2) on the primary rectal tumor with semi-annular morphology and extension from hours 2 to 5 were performed. The distal margin of the tumor was 90 mm from the external anal orifice. The tumor extended into the caudal skull for 13 mm and had a maximum axial thickness of 5 mm. The ECT treatment was performed using an expandable divergent electrode with 10 degrees of divergence, a length of 20 cm, and a 5 mm shaft (STINGER IG0E821).

### 2.4. Third LARC Case Description

**Figure 3:**
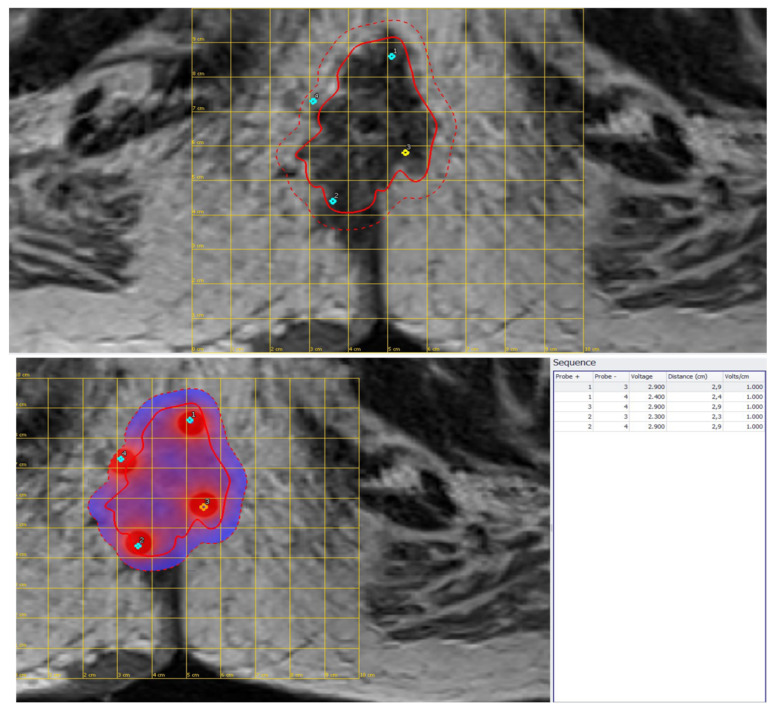
Preoperative planning of third LARC case effected using MR images.

A 50-year-old male with recurrence after long course neoadjuvant therapy and total mesorectal excision (TME). The treatment was performed with four single needles in a variable geometry (Figure 3), defined using a preoperative planning tool (Pulsar, IGEA Ltd., Modena, Italy). A single needle has 30 mm of active part and 20 cm of length. Moreover, one fixed geometry electrode with hexagonal configuration (EPSA, IGEA Ltd., Modena, Italy) was used to treat the patient. Multiple insertions to the subcutaneous recurrence part of the perianal region were performed.

## 3. Results of Included Cases

### 3.1. First Case

**Figure 4:**
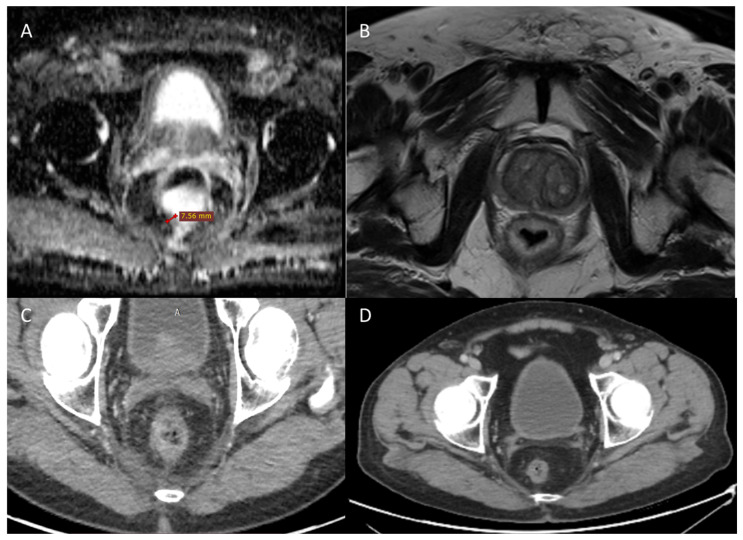
MR and CT images (**A**,**B**) before treatment of first case and CT images at 6 and 9 months after ECT treatment in (**C**,**D**), respectively.

The ECT treatment was determined accurately without current alarms during electric pulse delivery, and the procedure was considered safe and feasible by clinicians. MRI and CT before treatment showed thickening of the rectal walls (about 8 mm) with residual inhomogeneity of the wall (Figure 4A,B). The parietal thickening of the rectum was substantially stable, as shown in the CT scan performed 6 months after ECT treatment (Figure 4C). The lesion still appears in stable disease nine months after ECT (Figure 4D). The patient reported pain reduction according to the VAS scale from 2 to 0.

### 3.2. Second Case

**Figure 5:**
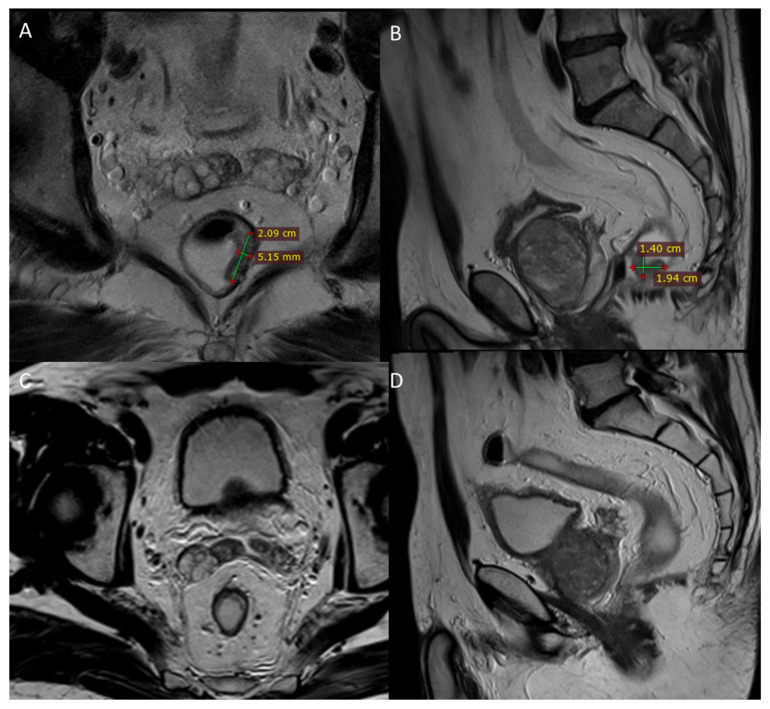
MR images (**A**,**B**) before treatment of second case and 2 months after ECT treatment (**C**,**D**).

The ECT treatment was determined accurately without current alarms during electric pulse delivery and the procedure was considered safe and feasible by clinicians. MRI and CT before treatment with ECT (Figure 5A,B) showed persistent rectal cancer after neoadjuvant therapy with semi-annular morphology and extension from 2 to 5 h. The neoplasm did not invade the mesorectal adipose tissue. MRI was performed 2 months after the ECT showed that the primary rectal tumor with semi-annular morphology and extension from 2 to 4 h had reduced in volume. The distal tumor margin was located 91 mm from the external anal verge, extending into the caudal skull for 24 mm, and appeared markedly hypointense on T2-weighted sequences due to extensive fibrosis. CT showed a slight residual thickening of the rectal walls on the side of apparent fibrotic content (Figure 5C,D). The patient reported pain reduction according to the VAS scale from 3 to 0.

### 3.3. Third Case

**Figure 6:**
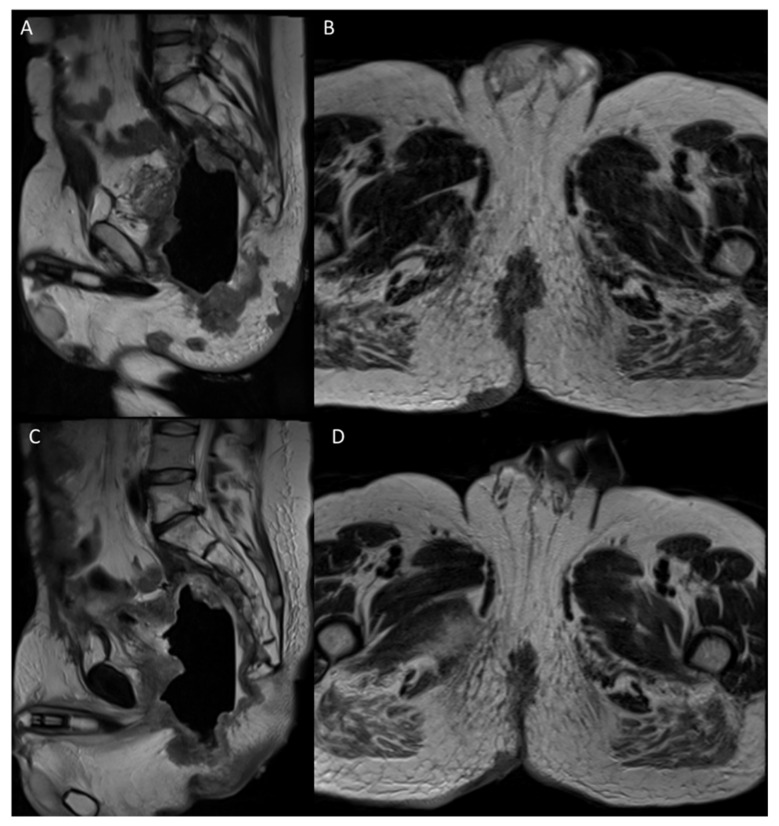
MR images (**A**,**B**) before treatment of third case and 6 months after ECT treatment (**C**,**D**).

The ECT treatment was determined accurately without current alarms during electric pulse delivery and the procedure was considered safe and feasible by clinicians. MRI was performed at a baseline (Figure 6A,B) and showed a large recurrence of the disease from the perianal skin region (also with distinct nodules), which travelled up along the perineum, leading anteriorly near the pubis, infiltrated, with fistula towards the wall anterior abdominal. Subsequently, the recurrence approached the sacrum, from which it was indissociable. The commitment of the presacral fascia and the right muscular structures was also appreciated, up to the iliac bifurcation. MRI 6 months after ECT treatment showed no significant morphostructural changes in the lesion that was considered stable disease (Figure 6C,D). However, the patient reported a reduction in pain on the VAS 7 to 4 scale and a reduction in bleeding.

## 4. Literature Review

TME is the gold standard in rectal cancer surgery and involves resection of the rectum together with surrounding fatty tissue [55]. Currently, 45–55% of LARC patients receive a combination of preoperative radiotherapy and chemotherapy (pCRT) in a neoadjuvant regimen before TME [56].

Since TME involves significant morbidity and complications, the need for conservative treatment strategies is strongly felt for patients with LACR and with a major response after pCRT with the advantage of reducing morbidity and preserving organs [57]. An improvement of survival seems to be related to the prolongation of the treatment interval [58,59,60].

Many advances have been achieved with ECT technique and due to the endoluminal approach; the difficulty of reaching the entire target lesion has been overcome in endoluminal cavities. The phase I clinical trial on the endoscopic treatment of advanced esophageal cancer revealed that the treatment is well tolerated and capable of inducing a reduction in mass [61]. No adverse events or complications were recorded in a phase II clinical trial underway in patients with colorectal cancer not suitable for surgery (https://clinicaltrials.gov/ct2/show/NCT03040180, accessed on 10 April 2021). Therefore, preliminary results of an endoscopic treatment for colorectal cancer highlight that the treatment is minimally invasive and ambulatory.

The advantage of this treatment is also evident for liver metastasis, whose indications of curative intent have increased in recent years. In fact, as demonstrated by population-based studies, 25–30% of patients diagnosed with CRC will develop liver metastases in the course of the disease, but despite oncological and surgical advances, only 25% of patients affected are candidates for resection [62,63]. Edhemovic et al. published the first experience of intraoperative colorectal liver metastases treatment [35]. 29 metastases in 16 patients were treated in 16 sessions of Variable Geometry Poration (VGP) and bleomycin. No serious adverse events were described and radiological evaluation showed 85% complete responses and 15% partial responses. Electrochemotherapy of colorectal liver metastases has proved a feasible, safe, and efficient treatment for metastases which are located near the main hepatic vessels and are not suitable for surgery or radiofrequency ablation [34,35,64,65].

A prospective, pilot study of feasibility, safety, and efficacy of intraoperative ECT for unresectable colorectal liver metastases was performed by Coletti et al. Linear or hexagonal needle electrodes according to an individualized pretreatment were used to perform ECT with an open liver resection approach [66].

A prospective study on 39 patients with metachronous colorectal liver metastases performed by Edhemovich et al. demonstrated the long-term effectiveness and safety of electrochemotherapy [67]. An objective response rate equal to 75% (63% CR, 12% PR) and median duration of the response equal to 20.8 months for metastases in CR and 9.8 months for metastases in PR was obtained. The best response was obtained for metastases smaller than 3 cm in diameter, while no difference was observed in metastatic location, i.e., metastases in central versus peripheral locations. Patients with a complete response after ECT showed a better progression-free survival compared to those metastases that had a partial response or progressive disease. However, there was no difference in overall survival for metastases smaller than 3 cm in diameter than for larger ones, and the median overall survival was 29.0 months [67].

## 5. Discussion

In this study, we present a prospective analysis of two patients with LARC subjected to neoadjuvant therapy and then to ECT, and of one patient subjected to ECT for colorectal local recurrence formed after neoadjuvant treatment and TME.

We reported that the ECT treatment is safe and feasible considering an endoscopic approach with a SILS port compared to a percutaneous approach using multiple needles in a variable geometry, planned with a preoperative planning tool before the treatment.

Preoperative planning was an important phase of ECT procedure when multiple needles in a variable geometry to ensure the complete coverage of the lesion were used. In these cases, the assistance of product specialists of the device was necessary (IGEA S.p.A., Carpi, Italy).

Furthermore, as preliminary results, we considered ECT in patients with LARC to be safe and feasible.

Moreover, the preliminary results showed stable disease in two of the three patients treated, while one patient achieved a complete response. The local control of disease was maintained in the patient follow-up. For each patient, a reduction in pain was observed and for the patient with local recurrence, a reduction in bleeding present before ECT was also achieved.

Therefore, our preliminary results showed that ECT could be used both in primary and secondary rectal cancer.

The treatment was safe and feasible both using an expandable electrode with fixed geometry, and multiple needles in a variable geometry adequately positioned considering a preoperative planning.

Moreover, in addition to the safety of procedures, the preliminary results could be considered promising in terms of the efficacy of ECT and in patients’ quality of life improvement (according to pain and bleeding reduction). 

The limits of this study were related to sample size. These results should be validated on a large group of patients since the results reported in this manuscript are only preliminary to a first experience in a cancer center. However, 35 patients with LARC undergoing neoadjuvant therapy should be enrolled in a randomized multicenter study [43] 12 weeks after the end of pCRT if they have had an important clinical response to undergo ECT with the intent to preserve the organ and guide towards a conservative surgery approach.

## 6. Conclusions

Preliminary results showed that ECT could be considered a safe and effective treatment in patients with a major clinical response or local recurrence after neoadjuvant therapy for LARC and could allow a reduction in pain and bleeding with a consequent improvement in quality of life.

## Figures and Tables

**Table 1:** Case descriptions and results.

Case N°	Description	Electrodes	Results
1	M 75 years old with major response was treated with ECT 12 weeks after chemo-radiation therapy	Expandable STINGER electrodes	The parietal thickening of the rectum was substantially stable. The lesion still appears in stable disease nine months after ECT. Pain reduction was obtained according to the VAS scale from 2 to 0.
2	M 66 years old with major response was treated with ECT 12 weeks after chemo-radiation therapy	Expandable STINGER electrodes	CT showed a slight residual thickening of the rectal walls on the side apparently of fibrotic content. The patient reported pain reduction according to the VAS scale from 3 to 0.
3	Male 50 years old with recurrence after long course neoadjuvant therapy and total mesorectal excision (TME) treated with ECT	Variable and fixed geometry electrodes	MRI 6 months after ECT treatment showed no significant morphostructural changes in the lesion that was considered to be stable disease. Patient reported a reduction in pain on the VAS 7 to 4 scale and a reduction in bleeding.

## Data Availability

Images are available at link https://zenodo.org/record/6538151#.YntzNOhBy3A (accessed on 12 April 2022).
